# Complementary and Alternative Medicine in Children with Type 1 Diabetes Mellitus

**DOI:** 10.4274/jcrpe.v3i3.27

**Published:** 2011-09-09

**Authors:** Belma Haliloglu, Pınar İşgüven, Metin Yıldız, İlknur Arslanoğlu, Müferet Ergüven

**Affiliations:** 1 Marmara University Faculty of Medicine, Department of Pediatric, Division of Pediatric Endocrinology, Istanbul, Turkey; 2 Göztepe Education and Research Hospital, Department of Pediatric Endocrinology, Istanbul, Turkey; 3 Düzce University Faculty of Medicine, Department of Pediatric, Division of Pediatric Endocrinology, Duzce, Turkey; 4 Göztepe Education and Research Hospital, Department of Pediatric, Istanbul, Turkey; +90 216 625 45 45-9109/ 9071 belmahaliloglu26@hotmail.com

**Keywords:** CAM, herbal medicine, type 1 diabetes mellitus

## Abstract

**Objective:** Complementary and alternative medicine (CAM) is increasingly utilized in adults and children for treatment of various conditions. Studies on CAM in diabetes have mainly focused on the adult population and its application in children has not been well established. The aim of this study was to examine the prevalence and characteristics of CAM use in Turkish children with type 1 diabetes mellitus (T1DM).

**Methods:** The information was acquired by a questionnaire completed by a face-to-face interview with the parents of children with T1DM.

**Results:** A total of 195 subjects (mean age: 14.02±4.7 years; F/M: 103/92) were included in this survey. Use of CAM was reported in 85 subjects (43.6%).  Herbal medicines were used in 64 subjects (75.3%). Sixty-nine subjects (81.2%) did not inform the diabetes specialist about CAM use. Thirty-eight subjects (44.7%) evaluated CAM as efficacious. Only 3 subjects (3.5%) interrupted the insulin injections to use CAM. No relationships were found  between CAM use and parental education or  insulin dose. There were significant correlations between CAM use and higher family income (p=0.027), urban residence (p=0.05), presence of complications (p=0.03), dissatisfaction with medical therapy (p=0.034) and prior CAM use among parents (p=0.001).

**Conclusion:** CAM use is a frequent practice among diabetic children, which is usually not shared with their physicians  and sometimes leads to cessation of medical treatment.

**Conflict of interest:**None declared.

## INTRODUCTION

Type 1 diabetes mellitus (T1DM) develops in genetically predisposed people as a result of the destruction of the  pancreatic beta cells caused by an autoimmune insulitis (1,2,3). At present, the incidence of T1DM is on the increase, while its age of onset decreases (4,5,6). Another worldwide trend noted in recent years is an increase in  use of complementary and alternative medicine (CAM). According to the National Institute of Health (USA), CAM is a wide health area which is outside the politically dominant health system in a society or culture in a specific time  interval and  includes health services, applications, methods, and their accompanying beliefs and theories (7). 

Also in Turkey, the use of CAM is increasing in both adults and children. We observed that many individuals  suffering from any kind of ailment tend to try various herbs, vitamins, antioxidant agents, yoga, meditation, bioenergy, acupuncture, aroma therapy and prayers in an effort to find a cure for their condition. 

The studies about the use of CAM in T1DM children are scarce. The objective of this study was to detect the methods and frequency of use of CAM in T1DM children and to compare those children with a group of controls with regard to variables such as age, sex, insulin requirement, presence of diabetic  complications, and sociocultural/ socioeconomic background. The study also aimed to explore the expectations of the families that motivated them to use these treatments.

## MATERIALS AND METHODS

The study was conducted on 200 T1DM patients under the age of 21 who were being followed in the Pediatric Endocrinology Department of Goztepe Education and Research Hospital. 

Before the research, an approval was obtained from the Ethics Committee of Goztepe Education and Research Hospital, as per their decision number 55/B, dated March 10, 2009. 

The families of the diabetic patients were given  information on the aims of the study and they were also told that they were free to leave the study at any time and to refrain from answering some of the questions.  CAM was defined as any treatment of a disease which is not included in the biomedical context. 

After written informed consent for interviewing and completing the questionnaire forms was obtained from  the patients’ parents, a questionnaire entitled “Use of  alternative medicine in T1DM patients” was filled out by interviewing each family face to face. 

 Five patients, whose questionnaire forms had been completed fully, were excluded from the study for being unable to answer the questions clearly. 

  The questionnaire included questions on demographic data (age, education level, socioeconomic status, place of residence), on frequency of use of CAM, method used, how it was used, reason for resorting to CAM, source of information on CAM, time of its initiation, association between CAM use and occurrence of complications, as well as questions on parents’ satisfaction with the medical treatment their children were receiving. 

 T1DM patients who were using CAM and those  who were not were compared with respect to the above-mentioned data.

  NCSS 2007& PASS 2008 Statistical Software (Utah, USA) program was used for the statistical analysis. During the assessment of the data of the study, besides the descriptive statistical methods (mean, standard deviation, frequency), the Student’s t-test was used for the  comparison of the quantitative data and normally  distributed parameters between the groups. Chi-square test and Fisher’s exact chi-square test were applied for the comparison of the qualitative data. The confidence interval of the results was 95% and the level of significance was taken as a p-value of less than 0.05. 

## RESULTS

The mean age of the patients (103F, 92M) was 14.0±4.7 years. 89.2% (n=174) of the mothers were  housewives and 76.4% (n=149) had attended school for at least five years.  52.3% (n=102) of the fathers were  workers and 68.2% (n=133) had attended school for at least five years. 48.2% (n=94) of the families had a monthly income between $611.62 and $1.223.24. 89.2% (n=174) were living in big cities. While no complications related to the diabetic state were reported in 90.8% (n=177) of the patients, 2.6% (n=5) had a concomitant chronic disease such as coeliac disease and autoimmune thyroiditis.  4.6% (n=9) of the parents expressed that they were not satisfied with the medical treatment their children were receiving and 29.7% (n=57) of the parents stated that they had used alternative treatment for themselves previously.  43.6% (n=85) of the parents had given alternative  treatment besides the medical treatment to their children, while the remaining 56.4% (n=110) had not. However, 71.3% (n=139) of the parents commented  that they would use these treatments if their children had a malignant condition. Herbal compounds, in particular thyme juice, cinnamon,  stinging nettle, aloe vera, were the most frequently mentioned substances used as CAM  (Tables 1 and 2). 

64.7% (n=55) of the parents stated that they applied CAM as a complementary method  supporting the medical treatment. 30.6% (n=26) reported that they got the  information from their friends and 29.4% (n=25) - from their families and close relatives. 31.8% (n=27) of the patients had begun to use CAM within 3 months of the onset of the  disease. 30.6% (n=26) stated that they had been using CAM for more than one year. Initiation of medical treatment was found to be delayed in 3.5% (n=3) of the patients  and 81.2% (n=69) of the parents did not share with their  doctors that they were using CAM methods. 44.7% (n=38) of the parents found CAM to be beneficial and 51.8% (n=44)  recommended these methods to others. 48.2% (n=41) of the parents who used CAM were still using the alternative  treatments at the time the questionnaire forms were filled out. 

 Although no statistically significant association was found between use of alternative treatment in children and  education level of parents, the rate of CAM use in big cities was markedly higher than that in small towns (p<0.05). It was also seen that as the level of income increased, the use of CAM in children increased (p=0.027) (Table 3). The use of alternative treatment was found to be significantly higher in children with diabetic complications or accompanying  autoimmune disease as compared to those with no  complications (p=0.030). The rate of CAM use was found to be considerably greater in children whose parents were not satisfied with the medical treatment and in children whose parents had previously used alternative treatment for  themselves than in those who were satisfied and those who had no experience of CAM (p<0.01 and  p<0.01, respectively). 

**Tables 1:**
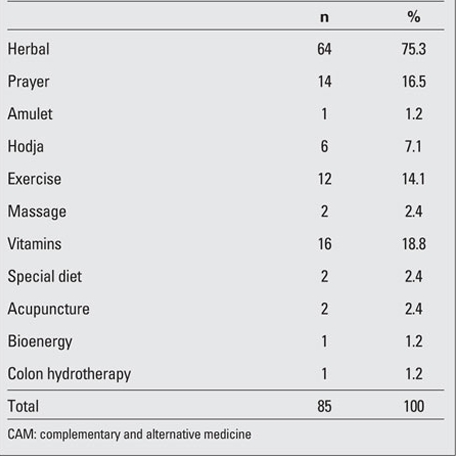
Table 1. The distribution of CAM methods

**2:**
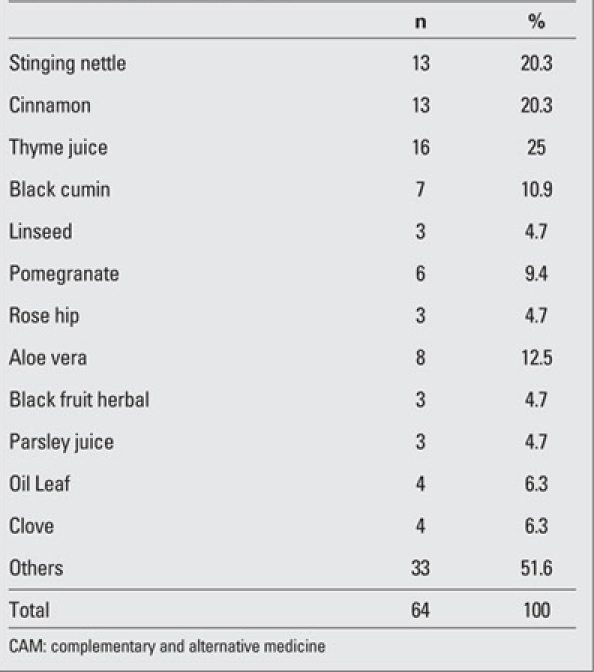
Table 2. The distribution of herbal CAMs

**Table 3:**
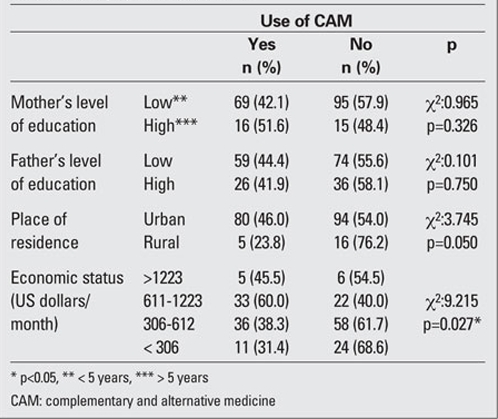
Table 3. Demographic characteristics

## DISCUSSION

According to previous studies conducted in Canada, Australia, Denmark and Turkey,  nearly half of parents 8,9,10,16)  and, to studies conducted in USA and New Zealand, approximately 20-40% of parents (11,12,13,14,15) reported using one CAM method sometime in their lives. 

 The number of studies on use of CAM in T1DM patients is limited. Miller et al (17) and Dannemann et al (18) reported that the frequency of CAM use in children with T1DM was 19% in the USA and 18.4% in Germany. Even though the rate in our study was found to be  relatively higher than that  estimated in other countries, this rate was compatible with the results of studies conducted in the general population and in children with chronic disease (16,19), as well as in children with T1DM (36) in Turkey. The high rate of CAM use in our patients may be due to the chronic nature of the disease, its labile course and the complexity of the medical treatments, which may have been the factors that led these parents to search for new ways of treatment to achieve better control (20,21).

In our study, the most frequently mentioned CAM method was administration of herbs. This finding was  consistent with the results of some of the studies reported from the USA (12,14) and of many studies from Turkey and  other  countries (16,19,22,23,24,25,26,27). Dannemann et al (18) found that herbs, homeopathy and vitamins were the most commonly applied CAM methods, while Miller et al (17) noted in their study that religious practices, use of herbs and vitamins were the most frequent ones. In our country, Arikan et al (36) reported that herbs were the most commonly used CAM method (59.6%), a finding also compatible with our results. Only 4 patients in our study had used homeopathy, naturopathy, or chiropraxy, which are methods more popular in other countries. The high cost of these practices may be the reason why they are not so preferred in Turkey.  As to the type of herb used, Dannemann et al (18) reported that cinnamon and aloe vera were the most  commonly used  ones, while Arikan et al (36) found that aloe vera, stinging nettle and mulberry were the herbs most frequently given to children with T1DM. In our study, the most commonly administered types were thyme juice, stinging nettle, cinnamon and aloe vera. Others have also reported that cinnamon and aloe vera are especially popular for the treatment of DM (29,30,31).  Although many studies showed that the use of CAM was directly proportional to the education level of the mothers (14,16,18,28) and the level of income of the  family (8,9,18), Pitetti et al (11) and Miller et al (17) did not find that these factors had a significant effect. In contrast, Arikan  et al (36) observed that the use of CAM decreased as the education level of the mother and the socioeconomic status of the family increased. It was also reported that the use of CAM by the parents themselves was a factor which increased the rate of use of CAM in their children 16,17,32,33). In our study, the use of CAM by the parents themselves was found to be directly proportional to the use of CAM in their children. Although we did not detect any correlation between CAM use and education level of the parents, the use of CAM was found to be significantly  higher in families having a high level of income and living in the big cities, a finding consistent with previous studies. We also found that when the parents were asked about using CAM if their children had cancer, the rate of use of CAM would increase from 43.6% to 71.3%.  This  result may be indicative of the parents’ view about DM as a treatable disease, even if it takes a lifetime. The risk of most complications of DM decreases with an adequate treatment, while cancer is perceived as a much more  mortal disease by the families.  The use of CAM was found to be significantly higher in patients with complications and in those with concomitant diseases. This finding may be due to increased anxiety  in the parents when faced with a complication or an  accompanying disease. In our study, religious beliefs were found to be  important factors encouraging the family  to use alternative treatment methods that were mostly applied to support the medical treatment. In our series, there were only 3 families who quit the medical treatment to use CAM. Dannemann et al (18) reported that the most commonly expressed  reasons for using CAM was “to try everything” and “their having fewer side effects”. 

As to source of information about CAM, we found that the majority of the parents had learnt about CAM from their friends and relatives. This finding is compatible with the results of similar studies in our country, while the  media and internet are reported as the most common sources of  information about the CAM methods in Western countries (16,34, 35). In our study, a few patients got the information from their doctors, but only 18.8% of the families shared this with their doctors. Ozturk et al (16) and Arikan et al (36) reported  similar results, while the rate estimated by Dannemann et al (18),  for sharing the information on use of CAM with their doctors, was much  higher. Also in Dannemann’s study, the parents stated that only 37.3% of the doctors advised their patients to leave the alternative treatment. The patients who did not tell their doctors about the use of CAM commented that their reason for not telling was that their doctors would not understand (18).   In this present study, when the parents were asked their opinion on the use of CAM, half of them said that they found them beneficial. This ratio was reported as 71.7% by Ozturk et al (16), as  53.8% by Arikan et al (36), and as 62.5% by  Dannemann et al (18). In all these studies, the issue of usefulness was evaluated by discourse with the families, i.e. subjectively, and was not based on objective criteria such as a decrease in the dosage of insulin or level of HbA1c.  Our study indicates that, similar to its practice in other chronic diseases, CAM is frequently used in children with T1DM.  As also shown in other studies from Turkey, herbs are the most commonly administered substances in our country. It also appears that when problems occur in the course of the disease, the rate of use of these substances increases. Studies are needed to determine their efficacy, safety, as well as the potential herb-drug or vitamin-drug interactions and to examine the side effects that can occur due to these interactions. In conclusion, pediatricians should communicate with the patients about the CAM methods and inform them about their benefits, harms and possible contraindications.
